# Recurrent symptomatic intraocular pressure spikes during hemodialysis in a patient with unilateral anterior uveitis

**DOI:** 10.1186/1471-2415-13-3

**Published:** 2013-02-06

**Authors:** Su-Ho Lim, Junhyuk Son, Soon Cheol Cha

**Affiliations:** 1 Department of Ophthalmology, Yeungnam University College of Medicine, Daegu, Republic of Korea

**Keywords:** Hemodialysis, Intraocular pressure (IOP), Trabeculectomy, Uveitis

## Abstract

**Background:**

The relationship between intraocular pressure (IOP) changes and hemodialysis has been evaluated for several decades. However, no report on an IOP rise in uveitis patients during hemodialysis has been previously documented. This report describes the case of an uveitis patient with repetitive IOP spikes associated with severe ocular pain during hemodialysis sessions, which resolved after glaucoma filtering surgery.

**Case presentation:**

A 47-year-old male with diabetes and hypertension had complained of recurrent ocular pain in the left eye during hemodialysis sessions. A slit-lamp examination showed diffuse corneal epithelial edema with several white keratic precipitates and inflammatory cells (Grade 3+) in the anterior chamber of the left eye. No visible neovascularization or synechiae were visible on the iris or angle. Topical glaucoma eye-drops and intravenous mannitol before hemodialysis did not prevent subsequent painful IOP spikes in the left eye. At the end of hemodialysis, IOP averaged ~40 mmHg. After trabeculectomy with mitomycin C in the left eye, his IOP stabilized in the low-teens (range, 10–14 mmHg) and no painful IOP spikes occurred during hemodialysis over the first postoperative year.

**Conclusion:**

We present a case of recurrent painful IOP spikes during hemodialysis in a patient with unilateral anterior uveitis unresponsive to conventional medical treatment prior to hemodialysis. To our knowledge, this is the first case report of repetitive symptomatic IOP rise during hemodialysis in an uveitic glaucoma patient. This case highlights the importance of the awareness of the possibility that IOP may rise intolerably during hemodialysis in uveitis patients with a compromised outflow facility.

## Background

Hemodialysis (HD) is an essential and common treatment for end-stage renal disease, and the ocular alterations associated with HD are wide ranging and include refractive changes, central corneal thickness changes, retinal nerve fiber layer thickness changes, and changes in intraocular pressure (IOP) [1-3]. The relationship between IOP changes and HD has been evaluated for several decades, and whereas some authors have found that HD does not affect IOP, others have concluded that it increases or decreases IOP [4].

Some have reported a significant rise in IOP during HD in eyes with a compromised aqueous outflow facility, such as, in eyes with narrow angles [5], neovascular glaucoma [6], or exfoliative glaucoma [7]. However, no report on an IOP rise in uveitis patients during HD has been previously published. Here, we present the case of an uveitis patient with repetitive IOP spikes associated with severe ocular pain during HD sessions, which resolved after glaucoma filtering surgery.

### Case presentation

A 47-year-old male with diabetes and hypertension had complained of recurrent ocular pain in his left eye during HD sessions. He had a history of kidney transplantation due to end-stage diabetic nephropathy seven years previously, but he had undergone regular HD three times weekly for two years due to post-transplant failure. Five years prior to this presentation, he had also undergone uncomplicated phacovitrectomy with intraocular lens implantation at another hospital, due to proliferative diabetic retinopathy in both eyes. Recently, he had become aware of a dull pain and blurred vision in his left eye during HD. His left IOP had reached levels of up to 54 mmHg over three weeks at a local eye clinic. Accordingly, he was referred to our glaucoma unit for management of uveitic glaucoma.

**Figure 1:**
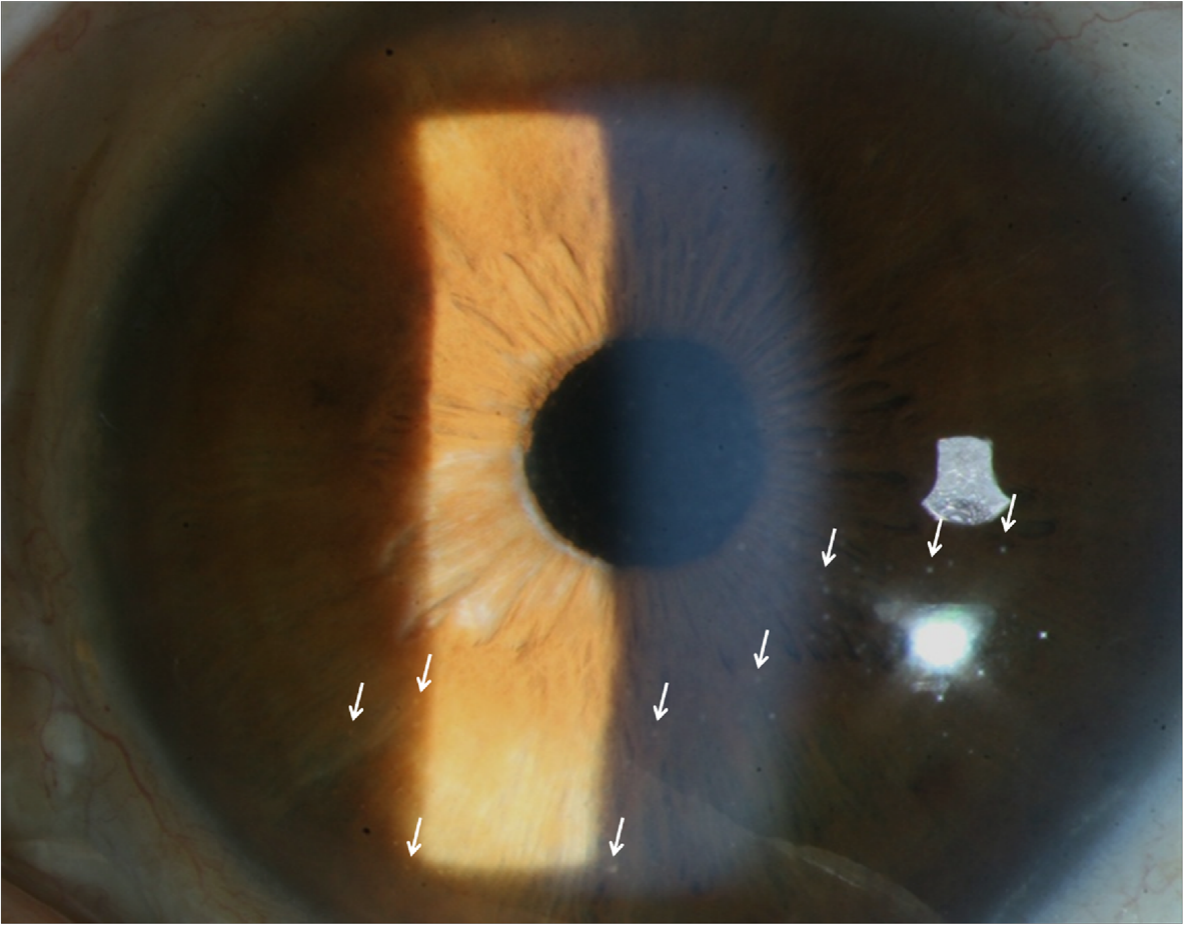
**Slit-lamp biomicroscopic findings in our uveitis patient.** The slit-lamp examination showed that inflammatory cellular reaction had subsided (Grade 1+) with small whitish keratoprecipitates at the lower half of the cornea (white arrow) 2 weeks after initiating topical 1% prednisolone acetate eye-drops. However, no visible angle or iris neovascularization was observed.

A slit-lamp examination showed diffuse corneal epithelial edema with several white keratic precipitates and inflammatory cells (Grade 3+) in the anterior chamber of the left eye (Figure 1). No sectorial iris atrophy or iris heterochromia developed during the follow-up period, which suggests herpetic keratouveitis or Fuchs’ heterochromic iridocyclitis. The gonioscopic findings showed a wide open-angle status (Grade 4, using Shaffer’s classification) and mild pigmentation (Grade 1 + ~2+) of the trabecular meshwork without angle neovascularization or peripheral anterior synechiae in both eyes. A more densely pigmented trabecular meshwork was observed in the left eye than in the right eye. Fundus examination revealed a clear vitreous, a slightly pale optic disc, a cup to disc ratio of 0.8, and well-applied photocoagulation scars in the left eye. The unaffected fellow eye did not show abnormal findings from the full ophthalmic examination, except for laser photocoagulation scar due to diabetic retinopathy. Serologic test for uveitis (include ESR, CRP, ANA, ANCA, HLA-B27, and VDRL etc.) and radiologic findings (chest X-ray and sacroiliac X-ray) were normal.

**Figure 2:**
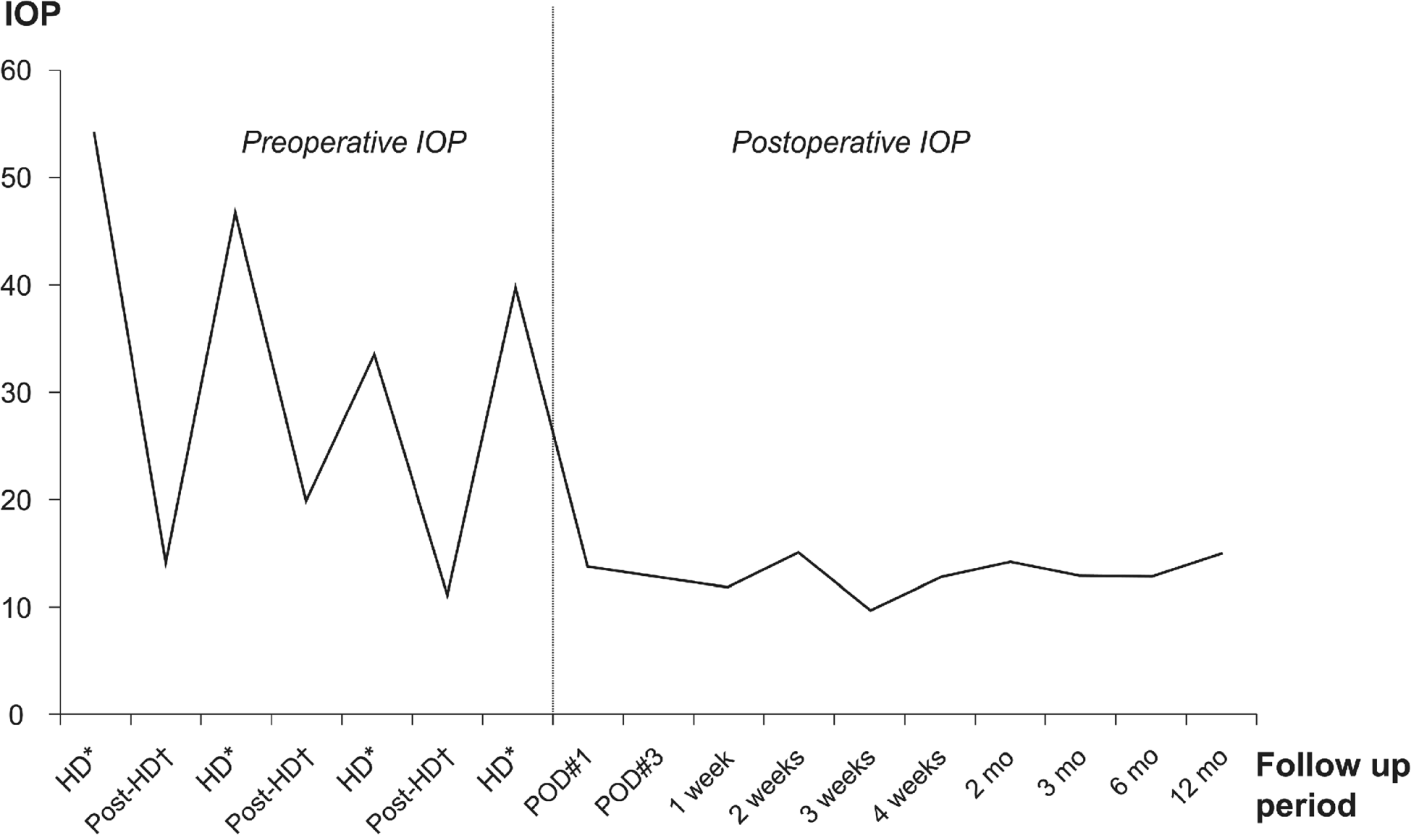
**Intraocular pressure fluctuation after hemodialysis in our uveitis patient.** *HD = Presenting IOP immediately after hemodialysis; †Post-HD = Presenting IOP at one day after hemodialysis; ^#^POD = Postoperative day. The mean IOP difference of the left eye before and after hemodialysis was 25 mm Hg, and this difference was statistically significant. Postoperatively, IOP remained stable in the normal range without medication.

The patient was initially treated with a fixed combination of dorzolamide-timolol eyedrops (twice a day), brimonidine eyedrops (twice a day), oral methazolamide (100 mg/day) to control his IOP, and 1% prednisolone acetate every two hours to manage inflammation. However, despite the glaucoma medications, recurrent IOP elevation with intolerable ocular pain occurred in the left eye about two hours after HD initiation forcing discontinuation and was not relieved by intravenous mannitolization. These events were subsequently repeated several times. Topical glaucoma eye-drops and intravenous mannitol before HD did not prevent subsequent painful IOP spikes in the left eye. At the end of HD, IOP averaged ~40 mmHg (range, 34–47). Accordingly, we decided on glaucoma filtering surgery. After trabeculectomy with mitomycin C in the left eye, his IOP stabilized in the low-teens (range, 10–14 mmHg) and no painful IOP spikes occurred during HD over the first postoperative year (Figure 2). Furthermore, the patient did not develop again signs of uveitis after the trabeculectomy during his hemodialysis.

## Discussion

We describe a case of recurrent painful IOP rise occurring about two hours after initiating HD in a patient with uveitis. To the best of our knowledge, this is the first case report of repetitive symptomatic IOP rise during HD in an uveitic glaucoma patient. Reports about IOP fluctuation during HD can be divided into three categories [4]; those that reported an IOP increase or decrease, and those reporting no change.

The proposed mechanism of IOP rise during HD involves a rapid fall in plasma osmolarity, resulting in an osmolar gradient between plasma and ocular tissue, causing water movement into tissue, increased aqueous production, and consequent IOP elevation [4]. Burn [8] provided a similar explanation and described it as being analogous to cerebral edema in disequilibrium syndrome. This mechanism was further supported by Ghaffariyeh et al. [9] who noted high vitreous urea rebound in a glaucoma patient with increased IOP during HD.

Then, why do IOP changes occur immediately after HD in uveitis patients and what is the clinical importance of these changes? It is possible that the compensatory mechanism of aqueous humor drainage is defective in uveitis patients. In particular, aqueous outflow might be reduced due to an increase in aqueous viscosity caused by inflammatory products and various cytokines, swelling or dysfunction of the trabecular meshwork, remodeling of the extracellular matrix, the obliteration of outflow channels, or synechial closure [10]. Tawara et al. [5] reported a remarkable increase in IOP during HD in eyes with impaired aqueous outflow as compared with eyes with normal aqueous outflow. Furthermore, Yoon et al. [11] reported that eyes with a pre-existing outflow obstruction had a high risk of IOP spikes during HD during the early postvitrectomy period.

Besides the compromised outflow facility, Rever et al. [12] reported that the anterior chamber depth decreased significantly during the HD. Moreover, Jaeger et al. [13] cited patients with narrow angles by gonioscopy who experienced an IOP rise during HD. In this context, gonioscopy is thought to be essential to the accurate diagnosis of IOP spikes related to HD.

A wide variety of inflammatory diseases are associated with ocular hypertension, such as Fuchs’ heterochromic iridocyclitis, glaucomatocyclitic crisis, herpetic keratouveitis, sarcoidosis, rheumatoid arthritis, and syphilis [14]. Therefore, careful patient evaluation is essential for accurate diagnosis and understanding of the pathogenesis of glaucoma. Especially, specific signs, patterns of IOP elevation, and past medical histories may also be evident in certain types of uveitis [14]. In our patient, the slit-lamp examination showed an intact corneal epithelium and some keratic precipitates without sectorial iris atrophy. Moreover, the gonioscopy revealed a wide open-angle status and mild pigmentation of the trabecular meshwork without angle neovascularization or synechiae. The results of the serologic test for syphilis, the HLA-B27, and the chest radiology findings were normal. Therefore, the authors diagnosed idiopathic acute anterior uveitis.

The prevention of marked IOP elevation during HD might be achieved using a combination of IOP lowering treatment and an appropriate HD technique [4], such as, slower urea removal or high-flux HD, which are associated with reduced urea rebound. Various medical treatment modalities have been used to manage IOP rise during HD, such as, topical and systemic carbonic anhydrase inhibitor, oral hyperosmotic agents, or intravenous mannitolization. However, these drugs cause electrolyte imbalances and metabolic acidosis, and although medication has been reported to be effective by some, in our patient, medication prior to HD did not prevent IOP elevation associated severe ocular pain. In addition, there are a limited number of surgical reports, such as, argon laser trabeculoplasty for exfoliative glaucoma [7], Ahmed valve implantation for neovascular glaucoma [15], and trabeculectomy for diabetic retinopathy [16] associated with IOP elevation during HD.

In a previous study of Strvrou and Murray [17], they reported that trabeculectomy might have a beneficial effect on the course of uveitis. They suggested that the improved aqueous outflow might allow the egression of inflammatory mediators from the anterior chamber. Furthermore, Weinreb [18] suggested that an anti-metabolite had a beneficial effect on intraocular inflammation, and some researchers [19] suggested that 5-FU might play a role in the management of anterior uveitis. Thus, trabeculectomy with adjunctive mitomycin C may theoretically improve uveitis and yield a successful surgical outcome.

## Conclusion

We present a case of recurrent painful IOP spikes during HD in a patient with unilateral anterior uveitis unresponsive to conventional medical treatment prior to HD. We hope that this case report makes physicians aware of the possibility of painful IOP elevation during HD in uveitis patients with a compromised outflow facility.

### Consent

Written informed consent was obtained from the patient for publication of this Case report and any accompanying images. A copy of the written consent is available for review by the Editor of this journal.

## Abbreviations

HD: Hemodialysis; IOP: Intraocular pressure.

## Competing interests

The authors declare that they have no competing interests.

## Authors’ contribution

SHL - participated in information gathering, literature search, data analysis, drafting of the case report, and final approval of manuscript. JS - participated in literature search, drafting of the case report and final approval of manuscript. SCC - conceived the idea, participated in information gathering, performed the surgery, data analysis, and approved the final manuscript. All authors read and approve the final manuscript.

## Pre-publication history

The pre-publication history for this paper can be accessed here:

http://www.biomedcentral.com/1471-2415/13/3/prepub
